# Co-identification of candidate regions associated with ovule number per ovary through QTL analysis and GWAS in *Raphanus sativus* L.

**DOI:** 10.1270/jsbbs.25016

**Published:** 2025-11-08

**Authors:** Jie Ji, Hui-Cong Xue, Xing-Yu Zhu, Hiroto Kobayashi, Ainan Tian, Kenta Shirasawa, Hideki Hirakawa, Nobuko Fukino, Masaya Yamamoto, Hiroyasu Kitashiba

**Affiliations:** 1 Graduate School of Agricultural Science, Tohoku University, 468-1 Aza-Aoba, Aramaki, Aoba-ku, Sendai, Miyagi 980-0845, Japan; 2 Kazusa DNA Res Institute, Kazusa-kamatari, Kisarazu, Chiba 292-0818, Japan; 3 Institute of Vegetable and Floriculture Science, NARO, 360 Kusawa, Ano, Tsu, Mie 514-2392, Japan

**Keywords:** *Raphanus sativus*, ovule number, seed number, QTL analysis, GWAS

## Abstract

Ovule number per ovary (ONPO) directly determines seed quantity in plants. In this study, two radish (*Raphanus sativus* L.) accessions exhibiting marked phenotypic variation in ONPO and seed number per pod (SNPP) were selected to generate bi-parental populations for quantitative trait locus (QTL) analysis. Additionally, genome-wide association studies (GWAS) on ONPO using 206 radish accessions were conducted. Through an integrated analysis of QTL mapping and GWAS results, a major common QTL was identified spanning a 0.6 Mb region on the terminal of chromosome 5. Based on genomic position, gene ontology, and expression analyses, *RsFLK* was highlighted as the primary candidate, along with two other selected genes *RsSGT* and *RsEMB3004*. Subsequent comparison of the *RsFLK* promoter sequences in the parental lines revealed unique InDels that may affect its expression, potentially contributing to the high-ONPO. These findings provide new insights into the genetic regulation of ovule number in radishes.

## Introduction

Seed number is a critical determinant trait for crop yield. In monocotyledonous plants such as rice (*Oryza sativa* L.) and wheat (*Triticum aestivum* L.), each ovary produces a single ovule that develops into one seed. Given this, identifying genes associated with seed number per panicle to enhance crop yields is of considerable importance (Lu *et al.* 2022). In contrast, research on dicotyledonous plants typically producing ovary containing multiple ovules such as Leguminosae and Brassicaceae, has focused on traits such as silique (seed pod) number per plant and seed number per silique (Jiang *et al.* 2020, Shi *et al.* 2009, Yang *et al.* 2017). However, interest in studying ovule development and improving upper seed number limits is increasing (Cucinotta *et al.* 2020). Radish (*Raphanus sativus* L., 2*n* = 18), a member of the Brassicaceae family, is an economically important vegetable crop cultivated worldwide not only for its edible taproot but also for the oil production potential of its seed. However, the number of seeds per pod (about 5) is very low (about 1/2 to 1/3) compared to that of *Brassica* vegetables, resulting in lower seed production efficiency in F_1_ hybrid varieties. Therefore, breeding for increased SNPP is expected to enhance seed yield and contribute to a more stable seed supply. Notably, considerable natural variation in SNPP has been observed among radish accessions, providing a valuable opportunity to investigate the underlying genetic mechanisms regulating seed and ovule numbers.

In most angiosperms, ovule development proceeds through several distinct stages (Barro-Trastoy *et al.* 2020). Ovules emerge from primordia on the placenta with primordium cells differentiating into specialized structures: the nucellus, chalaza, and funiculus. As development continues, integuments envelop the nucellus while the embryo sac and mega-gamete mature into the developed ovule. In *Arabidopsis thaliana*, the hormone-genetic networks governing the above-mentioned processes are extensively studied (Cucinotta *et al.* 2014, Schneitz 1999, Yang and Tucker 2021). For instance, during early development, the transcription factor *AINTEGUMENTA* (*ANT*) positively promotes ovule primordia outgrowth, while transcription factors *CUP-SHAPED COTYLEDON1* (*CUC1*) and *CUC2* function along the elongating primordia to establish proper ovule boundaries (Elliott *et al.* 1996, Ishida *et al.* 2000). Additionally, plant hormones, including auxins, cytokinins, brassinosteroids, and gibberellins, were also reported to regulate ovule number, size, and shape by modulating primordia formation and development (Barro-Trastoy *et al.* 2020).

Theißen *et al.* (2016) proposed a refined ABCDE model positioning ovules as the fifth floral whorl (Angenent and Colombo 1996), with ovule identity determined by proteins belonging to C, D, and E classes. In this context, recent studies on *hua-pep* mutants demonstrated reduced C and D class gene expression alongside ectopic expression of the A class gene *APETALA1* (*AP1*), leading to homeotic transformation of ovules (Rodríguez-Cazorla *et al.* 2020). Notably, either increasing the dosage of the C function gene *AGAMOUS* (*AG*) or inactivating A function genes *AP1* or *AP2* partially rescues ovule identity, indicating that coordinated activities of C and D class genes counteract A class gene effects—a finding consistent with the A-C antagonism observed in the ABC model.

Ovule and seed number traits are considered quantitative for plants such as *Brassica napus*. To identify quantitative trait-associated loci, QTL analysis and GWAS are widely employed as representative (typical) approaches with both methods generally requiring large population sizes, high marker densities, and precise phenotypic data to enhance detection power. Consequently, multi-location and multi-year trials are required to ensure robust results. However, for certain species and traits that are laborious to propagate and measure or for detecting minor-effect loci, integrated approaches combining QTL analysis, GWAS, and RNA-seq can be employed to cross-validate findings (Gelli *et al.* 2017, Han *et al.* 2018).

With advances in high-throughput next-generation sequencing (NGS) technology, it is now feasible to assemble high-quality genomes across diverse crop species. In population genetics, reduced representation sequencing (RRS) allows simultaneous genotyping of hundreds to thousands of samples, facilitating comprehensive molecular investigation of target traits. Among RRS techniques, restriction enzyme-based RAD-seq (Baird *et al.* 2008) and ddRAD-seq (Peterson *et al.* 2012) are widely used. More recently, PCR-based methods such as MIG-seq (Suyama and Matsuki 2015) have been developed to amplify genome-wide inter-simple sequence repeat (ISSR) regions. Building on this, a derivative method named dpMIG-seq (Nishimura *et al.* 2024) was established, in which primers partially containing degenerated sequences were used to increase the number of amplified loci. Both methods have been successfully applied as cost-effective alternatives for QTL mapping in wheat (Nishimura *et al.* 2022) and rice (Nishimura *et al.* 2024). However, the efficiency of these methods requires further validation in cross-pollinating plants.

To dissect the genetic basis of ovule number, this study investigated diverse radish germplasm exhibiting substantial variation in ONPO and SNPP. A significant positive correlation between these two traits was confirmed, and a major QTL for ONPO was mapped to the terminal region of Rs 5, corresponding to chromosome number 5 according to the nomenclature proposed by Shirasawa and Kitashiba (2017). Subsequent analysis of genes within this region selected three potential genes, especially *RsFLK*, for the specific InDels sequence in its promoter that may regulate ONPO. These findings lay a foundation for future functional studies and offer a promising molecular marker for radish breeding.

## Materials and Methods

### Plant materials

Two independent F_2_ populations (Year 2022, n = 146, Year 2023, n = 125) were developed from crosses between the radish accessions ‘Kameido’ (‘*K*’, JP27099, Genebank Project of NARO) and ‘RAPSAT-40’ (‘*R40*’, Tohoku Univ. *Brassica* Seed bank) for QTL analysis.

Additionally, a total of 206 radish accessions, representing diverse geographical regions (Japan, China and Korea, and South and Southeast Asia) were used for GWAS. These germplasms were sourced from the National Agriculture and Food Research Organization Genebank (Japan) and Tohoku University *Brassica* Seed Bank (https://sites.google.com/dc.tohoku.ac.jp/pbreed/brassica-seed-bank) as listed in Supplemental Table 1.

### Growth conditions and investigation of ONPO

For QTL analysis, F_2_ seeds obtained from F_1_ plants by crossing different parental individuals were pre-germinated and transplanted into small pots in the spring of 2022 and 2023. The plants were vernalized in growth chambers at 4°C, 12 h/12 h photoperiod for 30 days and subsequently transferred to the greenhouse located at the Graduate School of Agricultural Science, Tohoku University (38° 16ʹ N, 140° 50ʹ E).

For ONPO investigation, at least eight representative ovaries per plant were sampled from buds one day before flowering. The ovaries were treated with 1 M NaOH at 50°C for one hour, followed by staining in 0.1% aniline blue solution (0.1 M K_3_PO_4_) for 30 minutes to enhance ovule visualization under the fluorescence microscope.

All accessions used for GWAS were cultivated in garden pots (80 cm × 45 cm × 40 cm) in a glasshouse at the same location in 2019 (from September 2018 to June 2019), with five replicates per accession. Both hand pollinations and inflorescence shaking methods were employed to ensure fertilization. For ONPO assessment, five representative ovaries per plant were sampled and processed using the same staining protocol as described above. Additionally, ten well-filled seed pods, harvested at least 30 days after fertilization, were selected for seed counting.

### NGS and data processing

Genomic DNA was extracted from fresh leaf tissues using the CTAB method (Murray and Thompson 1980). MIG-seq (Suyama and Matsuki 2015) and dpMIG-seq (Nishimura *et al.* 2024) libraries were constructed for genotyping. The two F_2_ populations and bi-parents were sequenced using the Hiseq X and Novaseq X platforms (Illumina, Inc., CA, USA) with samples from different projects in Year 2022 and Year 2023, respectively. For GWAS, raw ddRAD-seq sequence data of 206 accessions were obtained from Kobayashi *et al.* (2020).

Adapter trimming and low-quality read removal were performed using Trimmomatic v.2.0 (Bolger *et al.* 2014). The same parameter was applied to reads obtained from MIG-seq and dpMIG-seq: HEADCROP:17 ILLUMINACLIP: dpMIGadapter.fasta: 2:30:10 SLIDINGWINDOW:4:15 LEADING:20 TRAILING:20 MINLEN:50. For ddRAD-seq, the parameter was: CROP:92 ILLUMINACLIP: ddRADadapter.fasta: 2:30:10 SLIDINGWINDOW:4:15 LEADING:20 TRAILING:20 MINLEN:90. FASTA files containing adapter sequences were prepared as dpMIGadapter.fasta and ddRADadapter.fasta, respectively. Processed reads were aligned to the radish reference genome (Yu *et al.* 2019) using BWA (Li 2011). SNP calling was conducted using Stacks (Catchen *et al.* 2013) for the F_2_ populations and GATK (McKenna *et al.* 2010) for the GWAS accessions. The nine chromosomes of radish were designated Rs 1 to Rs 9 following the nomenclature established by Shirasawa and Kitashiba (2017).

### QTL mapping

High-quality SNPs were filtered using vcftools with the parameter --minDP 10 (Danecek *et al.* 2011), followed by PLINK using --geno 0.2 and --mind 0.2~0.3 (Purcell *et al.* 2007). Genetic linkage maps for both F_2_ populations were constructed using the R package ‘onemap’ (Margarido *et al.* 2007) by incorporating chromosomes and position information from the reference genome. QTL analysis was performed in IciMapping (Meng *et al.* 2015) using the Inclusive Composite Interval Mapping (ICIM) method with a permutation-based LOD threshold (n = 1,000).

### Population structure and GWAS

Two complementary genome-wide association approaches were employed: single-locus GWAS (SL-GWAS) using GEMMA with an internal kinship matrix as a covariate (Zhou and Stephens 2012), and multi-locus GWAS (ML-GWAS) using an R package with principal components as covariates (Khan *et al.* 2019). SNPs exhibiting LOD scores ≥3.0 were identified as quantitative trait nucleotides (QTNs) associated with ONPO.

### Marker development and validation

Sequences harboring significant and non-significant ONPO-associated SNPs by GWAS were extracted and validated using Sanger sequencing (Sanger *et al.* 1977). PCR-RFLP markers were designed to detect polymorphisms between parental lines and within F_2_ populations. PCR products were digested with restriction enzymes and genotyped using polyacrylamide gel electrophoresis (PAGE). Primer sequences are provided in Supplemental Table 2.

### Gene annotation and Gene Ontology (GO) analysis

Genes within the major QTL interval and at QTNs were retrieved from the submitted genome annotation of the ‘WK10039’ reference. Functional annotation of these genes was subsequently performed using EggNOG-mapper v2.1.12 (Huerta-Cepas *et al.* 2019) based on the EggNOG v5.0 database.

### Gene expression analysis

To assess the expression levels of genes selected by Gene Ontology (GO) analysis, total RNA was extracted from immature ovaries at the ovule-identity stage, 6 to 7 days before flowering (corresponding to the 8 to 9 floral stage in *Arabidopsis* as defined by Schneitz *et al.* (1995)) using TRIzol (Invitrogen, CA, USA). RNA samples were treated with RQ1 RNase-Free DNase (Promega, WI, USA) to eliminate genomic DNA contamination. First-strand cDNA was synthesized using PrimeScript^TM^ RT Master Mix (TaKaRa, Japan) and quantitative PCR (qPCR) was performed using THUNDERBIRD^TM^ SYBR^TM^ qPCR Mix (TOYOBO, Japan) on a CFX96^TM^ Real-Time PCR Detection Systems (BIO-RAD) following standard protocols. Three biological and technical replicates were analyzed for the selected genes. Relative mRNA levels were normalized to glyceraldehyde-3-phosphate dehydrogenase (*GAPDH*). Primer sequences are listed in Supplemental Table 3.

### In-Fusion cloning and analysis of the *RsFLK* promoter region

The promoter region of *RsFLK*, defined as approximately 500 bp upstream of the transcription start site, was amplified by PCR from the two parental lines. The target PCR fragments were subsequently cloned into the pCAMBIA2300 plasmid (Addgene, MA, USA) using FastGene^TM^ PlasmidMini Kit (Nippon Genetic, Japan). Positive recombinant clones were screened by *Kpn*I digestion, and plasmid DNA was purified for Sanger sequencing (Supplemental Table 4).

Genomic sequences of *RsFLK* from multiple radish accessions (Supplemental Table 5) were retrieved via BLAST (Camacho *et al.* 2009) and aligned using SnapGene software (https://www.snapgene.com). Promoter sequences of *RsFLK* were independently extracted from each accession to predict conserved cis-regulatory motifs using the PlantCARE database (Rombauts *et al.* 1999). The motif distribution patterns were visualized using TBtools (Chen *et al.* 2023).

### Genome synteny analysis

Genome-wide synteny analysis among three radish genomes: ‘Sakurajima’ (Shirasawa *et al.* 2020), ‘WK10039’ (Yu *et al.* 2019) and ‘Xin-li-mei’ (Zhang *et al.* 2021) was analyzed using MCScanX (Wang *et al.* 2012). Synteny plots were generated using SynVisio (Bandi and Gutwin 2020) with the following parameters: -s 10 -e 1e-10 -m 10 -k 100 -g -2 -w 10.

### Statistical analysis

All statistical analyses were conducted using IBM SPSS Statistics 27 (IBM Corp., NY, USA). Continuous variables were presented as mean ± standard deviation (SD). Student’s *t*-test was used to assess differences between two groups. One-way ANOVA followed by Tukey’s test was applied to compare multiple groups. Correlation analysis between ONPO and SNPP was conducted using Pearson’s correlation coefficient. The level of significance was set at *P* < 0. 05 and *P* < 0.01.

## Results

### Phenotypic variations in seed number and ovule number

**Fig. 1.**
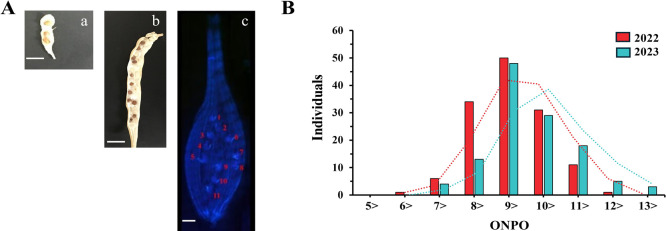
ONPO in the bi-parental lines and their derived F_2_ population. A. Anatomical observations SNPP in (a) ‘Kameido’ and (b) ‘RAPSAT-40’, and (c) ONPO observed after aniline blue staining. B. Distribution of ONPO in the F_2_ populations over two years. Scale bars: 1 cm (a, b), 130 μm (c).

The F_2_ progeny derived from crosses between ‘Kameido’ (average of ovule number 5.5 ± 1.0) and ‘RAPSAT-40’ (average 13.1 ± 2.6) exhibited normal distributions for ONPO in both the spring of 2022 and 2023, indicating trait suitability for QTL analysis (Table 1, Fig. 1).

**Fig. 2.**
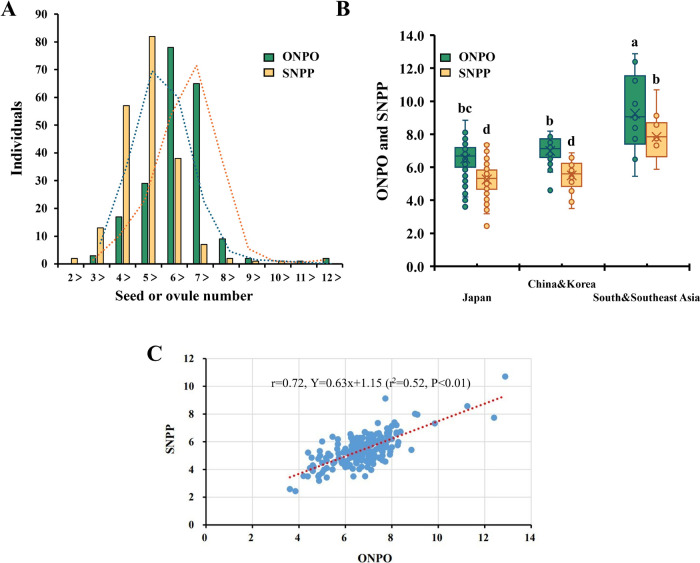
ONPO and SNPP of radish accessions from Japan, China and Korea, and South and Southeast Asia in 2019. A. Histogram showing the distribution of ONPO and SNPP. B. Differences in ONPO and SNPP among accessions from different regions (*P* < 0.01, ANOVA). C. Correlation between ONPO and SNPP, *r* = 0.72, *r*^2^ = 0.52, *P* < 0.01.

In addition, the phenotypic data of ONPO from 206 accessions and SNPP from 203 accessions were successfully obtained in 2019. The ONPO values ranged from 3.6 to 12.9, while the SNPP values ranged from 2.4 to 10.7 (Table 1). A comparison of the average trait values among geographic groups showed that accessions from South and Southeast Asia have significantly higher ONPO and SNPP than those from Japan, China, and Korea (Fig. 2B). Pearson’s correlation analysis revealed a moderate positive correlation between SNPP and ONPO (*r* = 0.72, *r*^2^ = 0.52) (Fig. 2C), suggesting that increasing of ONPO could substantially contribute to enhancing seed production in radish.

### Linkage map construction

To maximize genome-wide SNPs collection, a comparative analysis focusing on polymorphism detection between MIG-seq (Suyama and Matsuki 2015) and dpMIG-seq (Nishimura *et al.* 2024), an improved version of MIG-seq, was conducted. Both methods were processed in the same NGS run to minimize output variations.

**Fig. 3.**
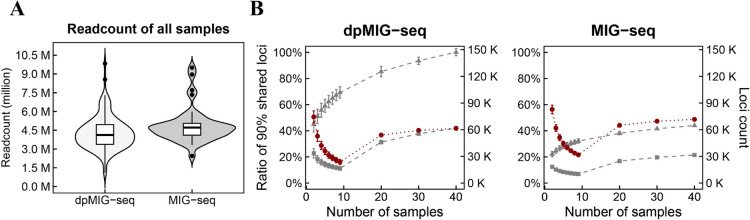
Performance comparison between MIG-seq and dpMIG-seq. A. Average read counts of MIG-seq and dpMIG-seq in 40 tested progenies. B. Number of total mapped loci and shared loci. The red circles, gray triangles, and gray rectangles indicate the mean ratio of shared loci, total loci count and shared loci count, respectively.

As a result, using any 40 F_2_ individuals, 194,683,649 raw reads (average 4,867,091 per progeny) for MIG-seq and 186,460,767 raw reads (average 4,661,519 per progeny) for dpMIG-seq were generated (Fig. 3A). As sample size increased, mapped loci increased proportionally, with dpMIG-seq consistently identifying 2.3-fold more mapped loci than MIG-seq, reaching approximately 150,000 and 64,000 loci, respectively (Fig. 3B). Meanwhile, polymorphism analysis (depth >5) between parental lines revealed 6,558 polymorphisms using MIG-seq and 8,509 using dpMIG-seq, with 2,758 shared polymorphisms constituting 22.4% of the total detectable variants (Supplemental Fig. 1). Additionally, average physical distances between polymorphisms were 50,482 bp for MIG-seq and 38,935 bp for dpMIG-seq, with maximum gaps of 1,601,087 bp and 1,854,245 bp, respectively. Notably, gaps in one method were frequently complemented by polymorphisms detected by the other method (Supplemental Fig. 2).

Based on the above data, for F_2_ population in 2022 and 2023, the dpMIG-seq method was employed and constructed two linkage maps comprising of 433 and 507 SNP markers, spanning 1,844.9 cM and 2,879.0 cM, with average marker intervals of 4.3 cM and 5.7 cM, respectively (Supplemental Fig. 3).

### QTL analysis for ONPO

QTL analysis for ONPO of the 2022 F_2_ population detected three significant QTLs exceeding the permutation-based threshold (1,000 iterations): two on Rs 5 and one on Rs 9, with LOD values of 4.2, 6.5, and 4.3, respectively (Supplemental Fig. 4, Supplemental Table 6). Similarly, the 2023 population detected six significant QTLs: three on Rs 5 and three on Rs 2, Rs 6, and Rs 7. No significant direct additive effects were detected between these QTLs.

Notably, the QTL signal *qON-5.2* identified on Rs 5 in 2022 lacked clear delineation at its terminal boundary due to the limited of available markers (Supplemental Fig. 5), necessitating development of additional markers to extend the genetic map and precisely define this QTL region.

### GWAS for ONPO

**Fig. 4.**
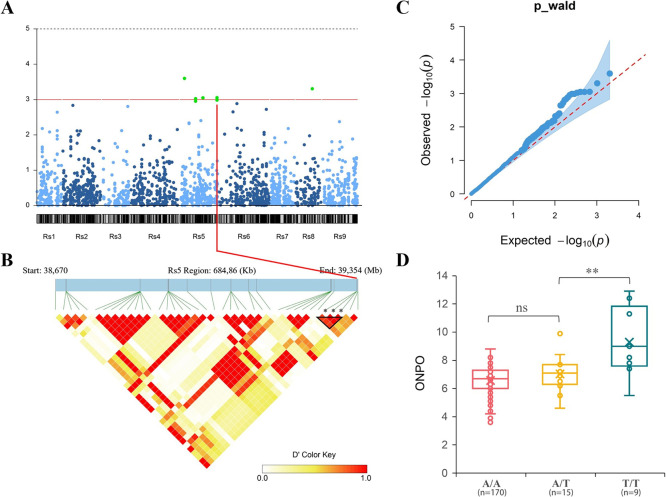
SL-GWAS results in the natural population. A. Manhattan plot showing the results of SL-GWAS for ONPO. B. Heat map of SNPs located at the terminal region of Rs 5. Asterisks indicate the positions of *q5.5*, *q5.6*, and *q5.7*. C. Quantile-quantile plot of the SL-GWAS results for ONPO-associated SNPs. D. Distribution of ONPO in 194 accessions grouped by the haplotype of the *q5.7* site. ‘**’ and ‘ns’ indicate statistically significant (*P* < 0. 01, ANOVA) and non-significance, respectively.

To complement our QTL findings, raw sequencing data was re-collected from Kobayashi *et al.* (2020), who previously conducted ddRAD-seq analysis across 510 geographically diverse radish accessions. Quality filtration of this data yielded 6,870 SNPs from 194 accessions suitable for SL-GWAS, identifying eight significant SNPs associated with ONPO (Fig. 4, Supplemental Table 7).

Of particular interest, the QTNs *q5.5*, *q5.6*, and *q5.7*, located 39,351,817 bp, 39,351,839 bp, and 39,351,845 bp on chromosome 5, were positioned approximately 4.6 Mb downstream of the last available marker (Rs 5: 34,739,830 bp) that delimits the right boundary of *qON-5.2* (Rs 5: 33,673,978–34,739,830 bp). These QTNs were also closely adjacent to a QTN at 39,354,468 bp detected by multi-locus GWAS (ML-GWAS) (Supplemental Fig. 6). Notably, apart from these three QTNs, only *q5.2* (Rs 5: 16,598,386 bp) was confirmed by ML-GWAS.

### Integration of GWAS-identified SNPs into linkage maps

Digestion of PCR products containing the *q5.7* locus with *Mse* I restriction enzyme revealed distinct banding patterns for parental lines ‘Kameido’ (‘*K*’) and ‘RAPSAT-40’ (‘*R40*’), and their F_1_ hybrid (Supplemental Fig. 7A). Subsequent genotyping of all progenies using this newly developed PCR-RFLP marker showed polymorphic bands in 133 of 134 progenies from 2022, with 32, 68, and 33 individuals showing banding patterns identical to ‘*K*’, F_1_, and ‘*R40*’, respectively (Supplemental Fig. 8). Similarly, genotyping of the 2023 F_2_ population revealed distributions of 31, 58, and 33 individuals matching the ‘*K*’, F_1_, and ‘*R40*’ patterns with clear genotype calls. Chi-square (χ^2^) analysis confirmed that marker segregation ratios in both F_2_ populations conformed to the expected 1:2:1 Mendelian ratio, validating the feasibility of adapting this newly developed PCR-RFLP marker (designated *Rs5_q5.7*). An additional PCR-RFLP marker (designated *Rs5_new2*) was further developed from a non-significant GWAS SNP located 0.17 Mb downstream of *q5.7* (Rs: 39,468,705 bp) (Supplemental Fig. 7B).

To evaluate whether these newly developed markers could be integrated into the existing genetic maps, we calculated the recombination frequencies with the terminal marker on Rs 5 and only marker pairs with a LOD score greater than 3 were considered closely linked. As a result, both markers were successfully incorporated into the original genetic map, extending the genetic distance of Rs 5 by 31.6 cM and 6.1 cM, respectively (Supplemental Fig. 7C). The genetic distances were estimated using the Kosambi mapping function (Kosambi 1943).

### Co-localization of ONPO-associated QTLs

**Fig. 5.**
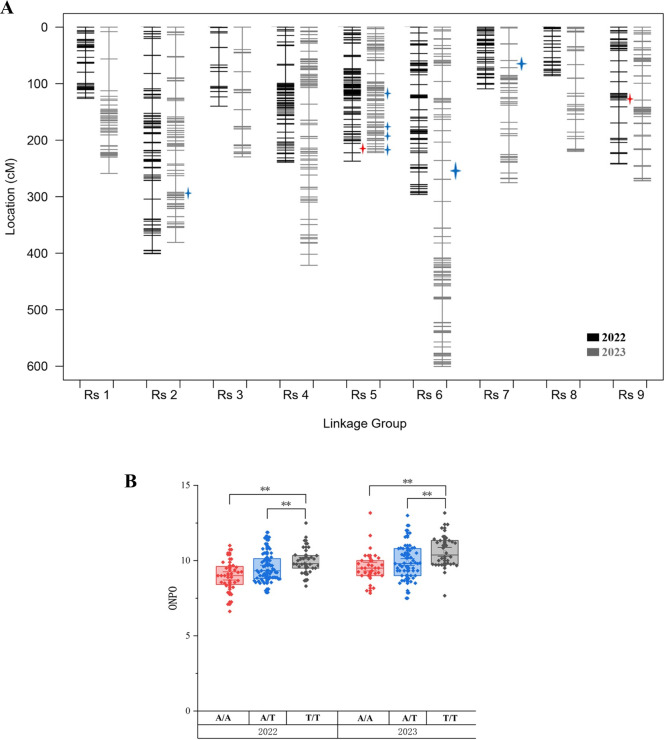
QTL mapping using updated linkage maps. A. Linkage maps after incorporating the two newly developed markers. Blue and red star marks represent QTL detected in 2022 and 2023, respectively. B. Distribution of ONPO in two F_2_ populations grouped by the haplotype at the *q5.7* site (*P* < 0. 01, ANOVA).

**Fig. 6.**
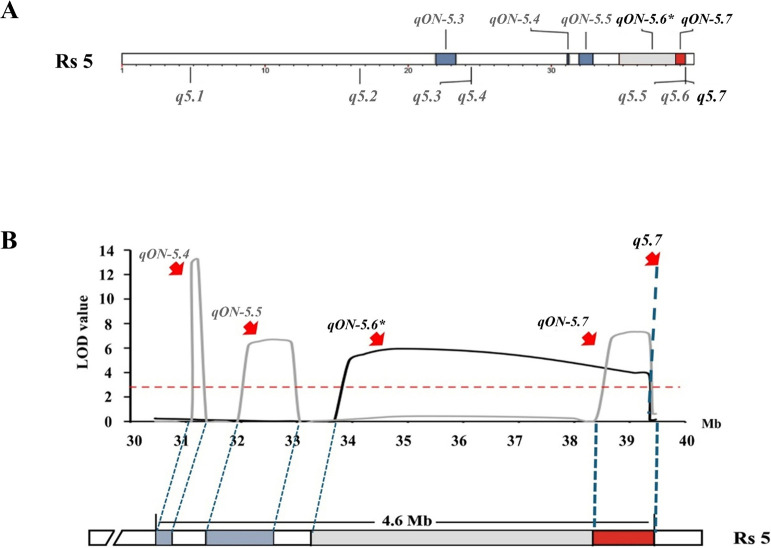
Identification of QTLs and QTNs associated with ONPO in this study. A. Physical positions of QTLs and QTNs detected by QTL mapping and GWAS on Rs 5. B. Co-localized QTL regions at the terminal of chromosome 5. Asterisk indicates the QTL *qON-5.6*, which was derived from *qON-5.2*. Gray and blue rectangles, as well as black and gray lines represent QTL intervals detected in 2022 and 2023, respectively. Red shaded areas indicate overlapping regions identified by all methods.

Using the expanded linkage maps, new QTL analyses were performed on the two-year F_2_ populations (Fig. 5A). It is worth noting that *qON-5.2*, previously having an undefined boundary at the Rs 5 terminal, now co-localizes with the *q5.7* site (hereafter designated as *qON-5.6*) (Table 2). Additionally, a novel QTL, *qON-5.7* was detected at the Rs 5 terminal in the 2023 population, sharing an approximately 0.6 Mb overlapping region with *qON-5.6* and encompasses *q5.7* (Fig. 6B). However, the peak of *qON-5.1* detected in 2022 fell below the significance threshold, possibly due to the incorporation of new markers as covariates in the analysis. No other overlap was observed between the QTL mapping and GWAS results (Fig. 6A).

### Annotation of genes controlling ONPO in candidate regions

A total of 145 annotated genes were identified within the overlapping interval of *qON-5.6* and *qON-5.7* (Rs 5: 38,663,009–39,351,845 bp), as well as the *q5.2* and *q5.7* site. Among these, *FLK* (*Flowering locus K*) emerged as the primary potential gene associated with ONPO, with the *q5.7* site located within its second intronic region, which was consistently validated by multiple approaches.

Further Gene Ontology (GO) analysis revealed 54.5% of the genes were associated with at least one GO term. Among these, terms related to biological processes (BP) such as development (GO:0032502), growth (GO:0040007), and reproductive processes (GO:0022414) were retrieved (Supplemental Fig. 9A). Finally, five additional genes (*RsEMB3004*, *RsSGT*, *RsCCT3*, *RsCLPS3*, *RsAGL3*) were picked based on their association with multiple GO terms potentially associated with ONPO (Table 3, Supplemental Fig. 9B).

### Expression analysis of the selected genes

**Fig. 7.**
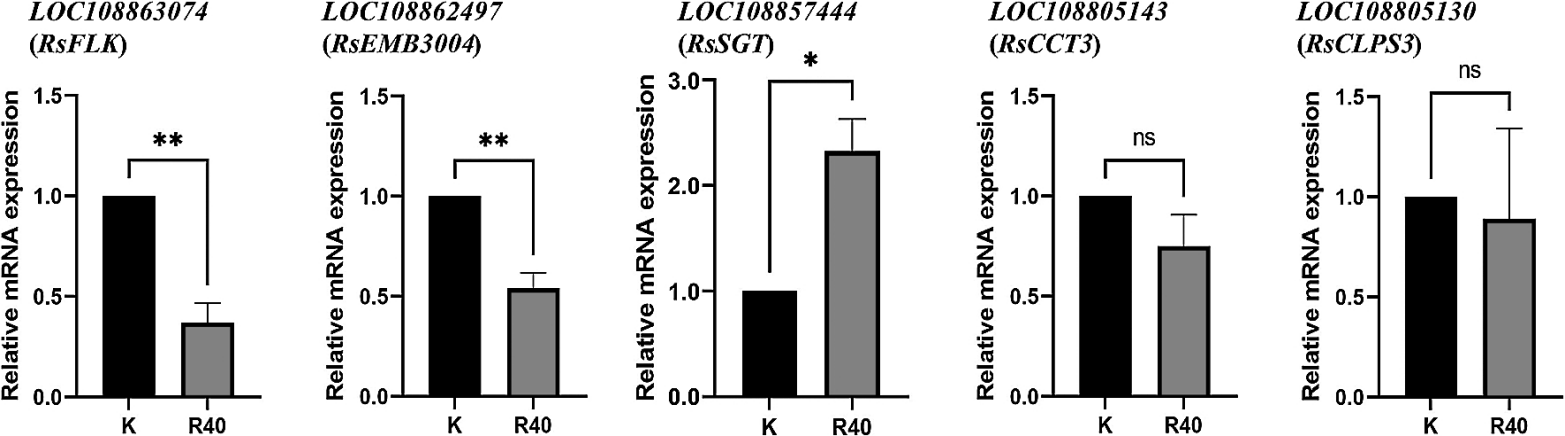
Relative mRNA expression of candidate genes normalized to *RsNADPH* in the two parental lines. Data represents the mean ± standard error (SE) of biological replicates. ‘K’ and ‘R40’ represent ‘Kameido’ and ‘RAPSAT-40’ while ‘*’ and ‘**’ represent statistically significant differences based on Student’s *t*-test (*P* < 0. 05 and *P* < 0.01, respectively).

To evaluate candidate gene expression levels, qRT-PCR was performed on immature ovaries with developing ovules to evaluate expression level differences between parental lines. The expression of Rs2_25309 could not be assessed due to poor amplification efficiency. Among the successfully analyzed genes, *RsFLK* and *RsEMB3004* showed markedly reduced expression in ‘RAPSAT-40’ (‘*R40*’), displaying less than 50% of the transcript levels observed in ‘Kameido’. Conversely, *RsSGT* demonstrated more than two-fold higher expression in ‘*R40*’. However, the expression levels of *RsCCT3* and *RsCLPS3* showed no significant differences between the parental lines (Fig. 7). These preliminary findings suggest promising directions for further investigation into the molecular mechanisms governing ONPO regulation.

### Unique InDels identified in the promoter region of *RsFLK*

Given the significant difference in the expression level of the primary candidate *RsFLK*, we investigated the promoter region as a potential factor responsible for this variation. BLAST and sequence alignment of the *RsFLK* sequence against the publicly available radish assemblies revealed a highly conserved gene structure (Supplemental Fig. 10). Notably, the ONPO-associated SNPs *q5.5*, *q5.6*, and *q5.7* within the second intronic region was observed in one rat-tail radish accession, exhibiting a genotypic haplotype identical to that observed in ‘RAPSAT-40’ (‘*R40*’).

**Fig. 8.**
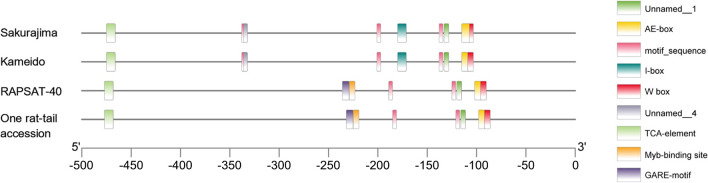
Comparison of conserved cis-acting regulatory elements within the promoter regions among three radish accessions and the two parental lines. Note that TATA-box and CAAT-box elements were excluded from the visualization due to their redundant presence in the promoter regions.

Subsequent comparison of the *RsFLK* promoter sequences among the radish accessions identified one insertion sequence harbors a MYB-binding site (CAACAG) and a GARE-motif (GTTGTCT) as well as two deletion sequences, located at the domain of I box (GTATAAGGCC) and unnamed-4 element (CTCC), respectively. These InDel variants were exclusively present in ‘*R40*’ and the rat-tail radish accession carrying the specific intronic haplotype (Fig. 8). Notably, *RsFLK* expression was significantly lower in ‘*R40*’ compared to ‘Kameido’, suggesting a possible association between the presence of these InDels and reduced *RsFLK* expression, which may contribute to the observed variation in ONPO.

## Discussion

In this study, we investigated the genetic basis of ONPO in radish (*Raphanus sativus* L.) through an integrated quantitative genetics approach. To comprehensively interpret our findings, we discuss the performance and applicability of the employed genotyping methods, the phenotypic characteristics of both ONPO and SNPP, as well as explore potential candidate genes for ONPO within the major QTL region.

In the present study, the dpMIG-seq method was employed for genotyping bi-parental populations. Unlike restriction site-dependent RAD-seq methods, dpMIG-seq uses a PCR-based approach with primers that can anneal not only to SSR regions but also predominantly to incomplete or non-SSR sequences (Nishimura *et al.* 2024). Consistent with Nishimura’s (2024) findings, compared to MIG-seq, our dpMIG-seq results demonstrated wider read coverage but lower depth. Statistical analysis also demonstrated dpMIG-seq method advantages in terms of number and distribution of common loci. However, despite expectations of higher marker yields given radish’s larger genome size compared to rice, only half the anticipated markers were obtained in parental lines. This reduced efficiency likely stems from radish’s self-incompatibility, which limits available homozygous SNP necessarily for constructing linkage maps. These findings suggest that combining dpMIG-seq with complementary methods, such as developing PCR-Restriction Fragment Length Polymorphism (PCR-RFLP) markers at specific sites (used in this study) or incorporating Simple Sequence Repeat (SSR) markers outside ISSR regions, can be beneficial when the number of detected SNPs is insufficient, even for materials of limited homozygosity.

Through the investigation of ONPO and SNPP in 2019, a correlation coefficient between them of 0.72 was observed. The complete correlation was not observed possibly due to the insufficiencies in post-flowering development processes, such as fertilization efficiency and zygote development, which influence final seed numbers (Yang *et al.* 2017). For the ONPO trait, several novel genomic regions were identified in this study. Particularly, a reproducible region including *qON-5.6*, *qON-5.7*, and *q5.7* verified by twice QTL analyses and single GWAS was detected at the terminal of Rs 5. This region is not identical to that previously reported by Hashida *et al.* (2013), in which the QTL was detected at Chr I (=Rs2, Shirasawa and Kitashiba 2017), located approximately 0.34 Mb downstream of *qON-2.1*, indicating the identification of a novel region associated with ONPO through the present study.

For the detected QTL region, GO analysis revealed several genes of interest in ovule development regulation. Among these, *CLPS3* encodes a nuclear protein involved in mRNA processing with known effects on flowering and ovule development in *Arabidopsis thaliana*. Overexpression of *CLPS3* promotes early flowering and elevates key ovule development gene expression, such as *CUC1* and *WUSCHEL* (*WUS*) (Xing *et al.* 2008). Additionally, the *CCT* complex plays a crucial role in folding cytoskeletal proteins, which are essential for plant growth and development, such as cell division, elongation, and environmental responses (Himmelspach *et al.* 1997). However, no significant expression differences were observed in these two genes between parental lines in this study, suggesting the existence of novel genes associated with ovules.

Additionally, differential expression patterns were identified in *RsSGT* and *RsEMB3004*. Mutant plants deficient in *SGT* exhibit reduced levels of glycosides, leading to observable defects, such as reduced seed size (DeBolt *et al.* 2009), while disruptions of *EMB3004* result in embryo or gametophyte lethality in *Arabidopsis*. Although these genes display promising expression differences, their direct roles in ovule identity determination are unknown and warrant further investigation. More recently, Yuan and Kessler (2019) identified a gene named *NEW ENHANCER of ROOT DWARFISM* (*NERDI*) in *Arabidopsis thaliana* directly regulating ovule numbers. Although no association with ONPO was detected for its radish ortholog in the present study, its genomic position has been determined in multiple radish reference assemblies (Shirasawa *et al.* 2020, Yu *et al.* 2019, Zhang *et al.* 2021) (Supplemental Fig. 11), also offering a potential target for future investigation.

Of particular interest is *RsFLK*, a homolog of *Arabidopsis FLK*, which was initially characterized as a flowering time regulator via its antagonistic interaction with *FLOWERING LOCUS C* (*FLC*) (Mockler *et al.* 2004). Subsequent studies clarified its role as a key component of the *HUA-PEP* post-transcriptional regulatory module, which modulates flowering time and floral morphogenesis by perturbing the expression of *AG*, D-class, and other downstream genes. In *Arabidopsis*, severe homeotic transformation of ovules into floral organs has been observed in *flk*-containing multiple mutants (Rodríguez-Cazorla *et al.* 2015, 2018). However, unlike the changes observed in mutants, the precise role of *RsFLK* in regulating ovule identity in naturally occurring radish variants remains unclear and may involve a mechanism distinct from the canonical *HUA-PEP* pathway. Sequence comparison of the *RsFLK* promoter revealed two deletion sequences and one insertion sequence exclusively present in ‘RAPSAT-40’ (‘*R40*’) and the rat-tail radish accession sharing the same intronic haplotype. Compared to the insertion sequence which harbors MYB-binding site and GARE-motif, we hypothesize that the loss of I-box or unnamed-4 element is more likely responsible for the reduced expression of *RsFLK*. Although the ONPO of the rat-tail accession (*Raphanus sativus* var. *caudatus*) remains unknown, its seed pod is generally longer like that of ‘*R40*’ (Yamagishi 2017), suggesting the higher ovule and seed numbers, as reflected by the mean ONPO in ‘RAPSAT-40’ (12.9 ± 1.8), which was notably higher than that in ‘Kameido’ (4.9 ± 0.9) and ‘Sakurajima’ (6.3 ± 1.9). However, additional ONPO data and expression level from the rat-tail radish and other accessions will be necessary to validate this hypothesis and clarify the potential regulatory role of these transcription factors in ovule development.

Collectively, by integrating two years of QTL mapping with GWAS, this study identified a major region associated with ONPO in radish. *RsFLK* was highlighted among the potential genes based on the associated results and relevant biological reports. Promoter sequence analysis of *RsFLK* revealed specific InDels, especially the loss of I-box and unnamed-4 element in ‘RAPSAT-40’-like accessions. Further studies will be needed to understand if these elements are essential in regulating *RsFLK* and consequently influence ovule development, even final seed number in radish. Other potential genes and QTLs/QTNs proposed in this study, though not further explored here, remain valuable targets for future research.

## Author Contribution Statement

JJ: conceptualization, formal analysis, investigation, writing—Original Draft, and Funding acquisition. HX: conceptualization and investigation. XZ: discussion of the results and methodology. AT: discussion of the results and methodology. H. Kobayashi: discussion of the methodology. NF: discussion of the materials and results. MY: discussion of the methodology and results. H. Kitashiba: discussion of the results, conceptualization, resources, supervision, writing—review & editing, and funding acquisition. All authors read and approved the manuscript.

## Supplementary Material

Supplemental Figures

Supplemental Table 1

Supplemental Table 2

Supplemental Table 3

Supplemental Table 4

Supplemental Table 5

Supplemental Table 6

Supplemental Table 7

## Figures and Tables

**Table 1.** Observation of phenotypes in two-year F_2_ populations and natural accessions in 2019

Trait	Year	Population	Number	Average ± SD	Range
SNPP	2019	Natural	203	5.4 ± 0.1	2.4–10.7
ONPO	2019	Natural	206	6.7 ± 1.3	3.6–12.9
	2022	F_2_	146	9.5 ± 1.0	6.6–12.5
	2023	F_2_	125	10.0 ± 1.2	7.5–13.2

**Table 2.** Positions and effect of QTLs for ONPO using newly integrated linkage map

Trait	Year	QTL	Chr	Position (bp)	LOD	Add*^a^*	PVE (%)*^b^*
ONPO	2022	*qON-5.6*	5	34,739,830–39,351,845	5.9	0.54	16.3
		*qON-9.1*	9	32,285,271–32,625,683	3.8	0.42	10.3
	2023	*qON-2.1*	2	28,972,458–29,027,762	4.9	0.45	4.5
		*qON-5.3*	5	21,931,759–23,318,329	6.3	0.56	6.5
		*qON-5.4*	5	31,101,504–31,215,648	13.2	0.05	15.5
		*qON-5.5*	5	31,929,817–32,903,439	6.7	–0.003	7.3
		*qON-5.7*	5	38,663,009–39,351,845	7.3	0.53	7.5
		*qON-6.1*	6	30,706,956–33,658,294	7.7	0.08	7.6
		*qON-7.1*	7	1,748,372–1,839,083	8.4	0.08	10.2

*^a^*: Additive effect of ‘*R40*’ alleles is shown. *^b^*: Percent phenotypic variation explained by a QTL.

**Table 3.** Summary of annotated genes involved in at least two GO terms along with *FLK*

Gene of *R. sativus*	Annotation gene name of *A. thaliana*	Annotation function of *A. thaliana*	GO term
*LOC108805130*	*CLPS3* (*CLP-SIMILAR PROTEIN 3*)	Floral development	DP, RP*^b^*
*LOC108805143*	*CCT3* (*CHAPERONIN CONTAINING T-COMPLEX POLYPEPTIDE-1 SUBUNIT 3*)	Protein folding	DP, G
*LOC108857444*	*SGT* (*STEROL GLUCOSYLTRANSFERASE*)	Seed mass	DP, RP
*LOC108862497*	*EMB3004* (*EMBRYO DEFECTIVE 3004*)	Embryo development	DP, RP
*LOC108863074*	*FLK* (*Flowering locus K homology domain*)	Floral development	DP
*Rs2_25309^a^*	*AGL3* (*AGAMOUS-LIKE 3*)	Floral development	DP, RP

*^a^*: hypothetical protein only has gene id. *^b^*: DP, RP, G represent development process, reproductive process, and growth, respectively.
