# Supplementary material for: Co-identification of candidate regions associated with ovule number per ovary through QTL analysis and GWAS in Raphanus sativus L
Source: Breed Sci. 2025 Nov 8;75(5):400–11. doi: 10.1270/jsbbs.25016 (PMC13129575; doi:10.1270/jsbbs.25016)
Supplement: Supplementary file 1 — Supplemental Figures [file 75_400_s1.pdf]

doi: 10.1270/jsbbs.25016

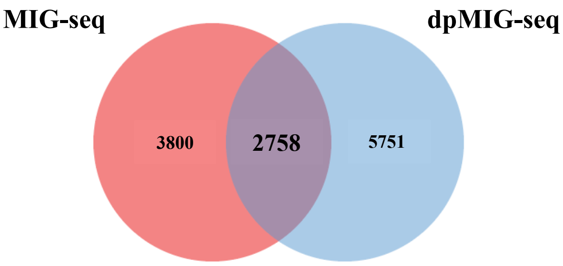

Supplemental Fig. 1 Polymorphisms detected via MIG-seq and dpMIG-seq in parental lines.

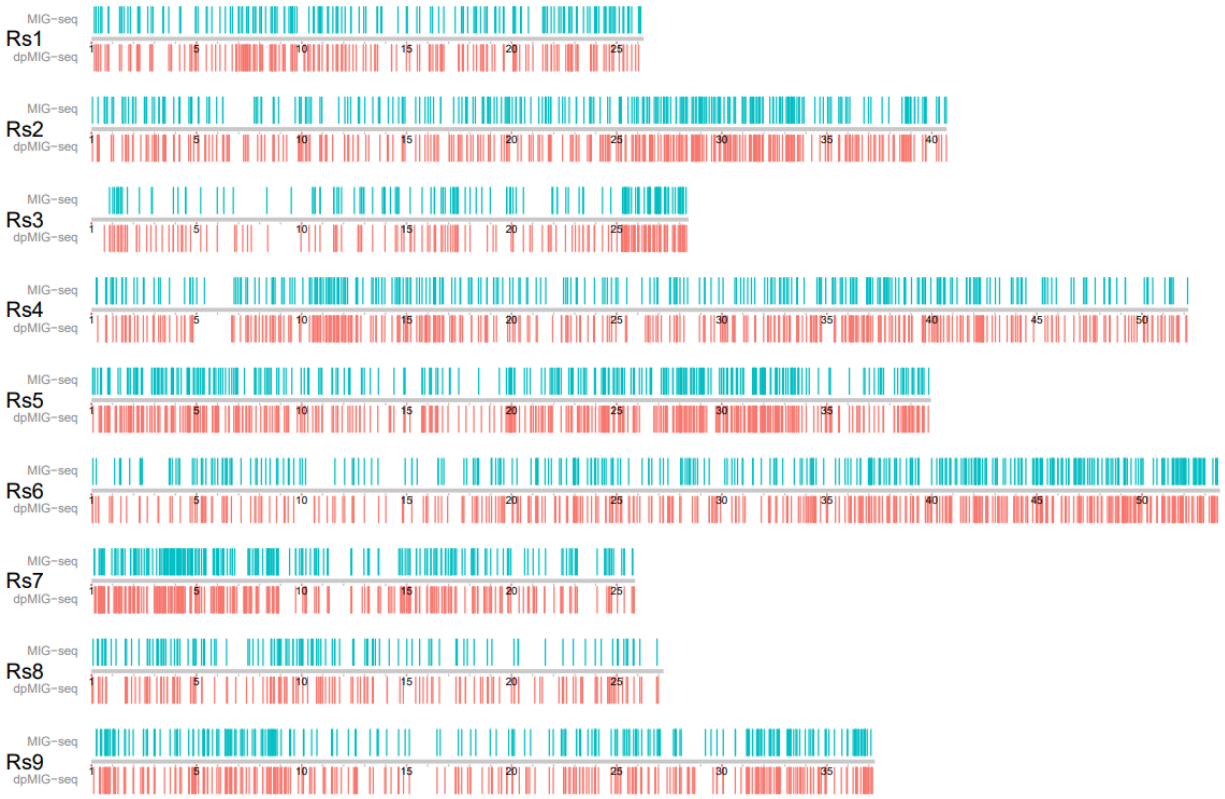

Supplemental Fig. 2 Distribution of polymorphisms detected in the parental lines using MIG-seq and dpMIG-seq with a 10,000 bp window size. The blue columns and red columns represent polymorphisms detected by MIG-seq and dpMIG-seq, respectively.

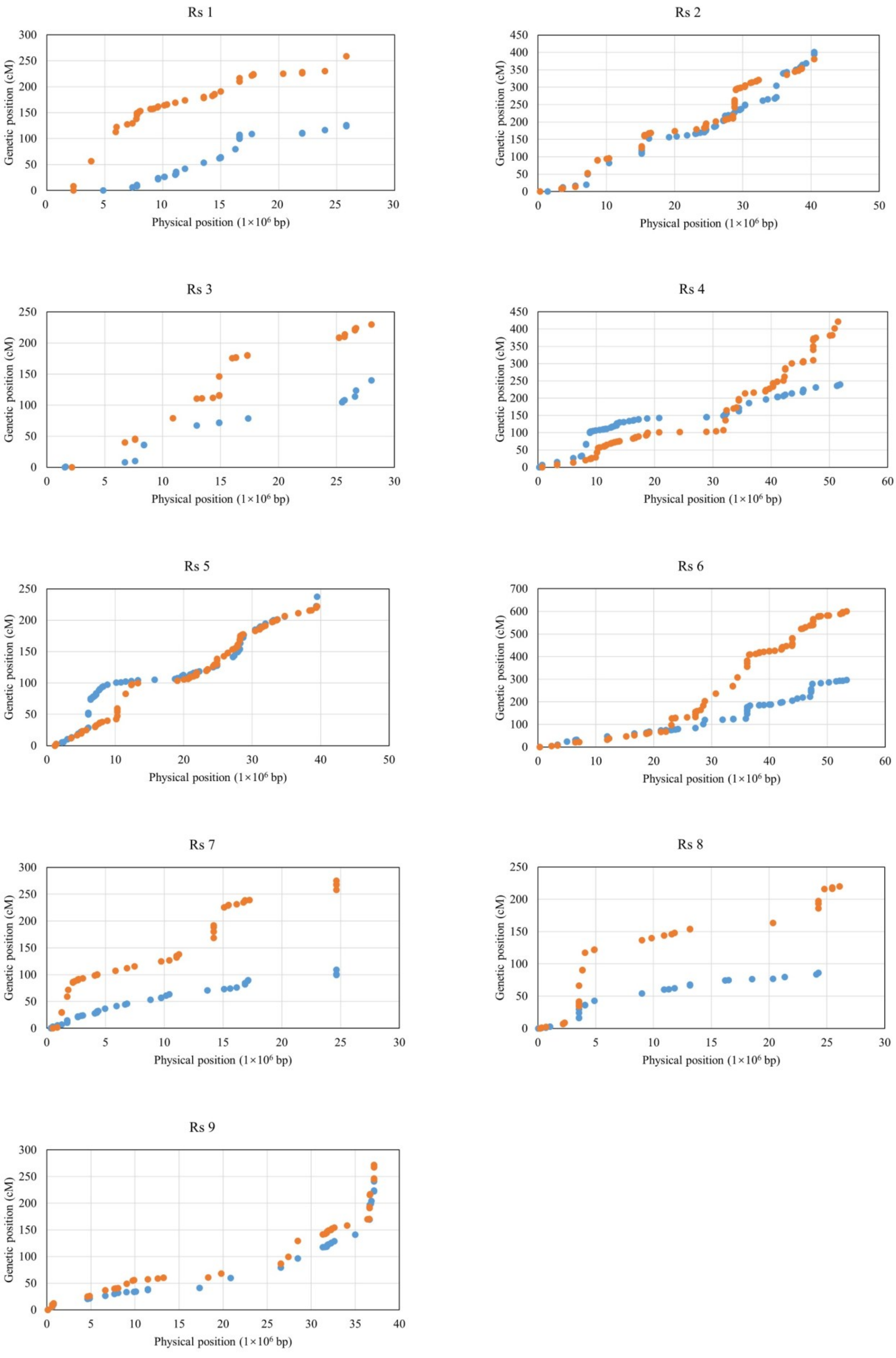

Supplemental Fig. 3 Physical and genetic positions of SNP markers used for constructing linkage maps in 2022 and 2023. Blue and orange dots represent SNPs retained in the F_2 populations of 2022 and 2023, respectively.

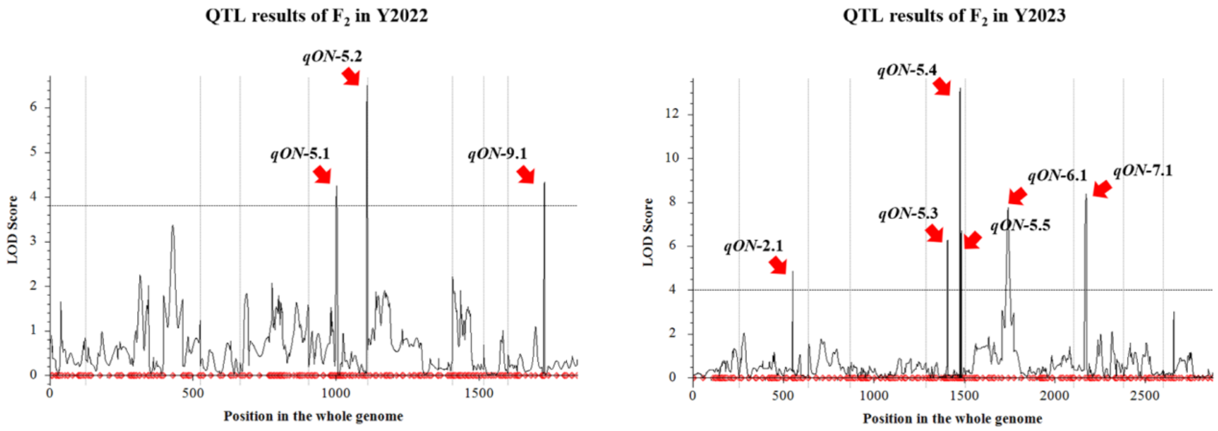

Supplemental Fig. 4. QTL mapping results for F_2 populations across two consecutive years. Red arrows indicate the peaks of QTL signals.

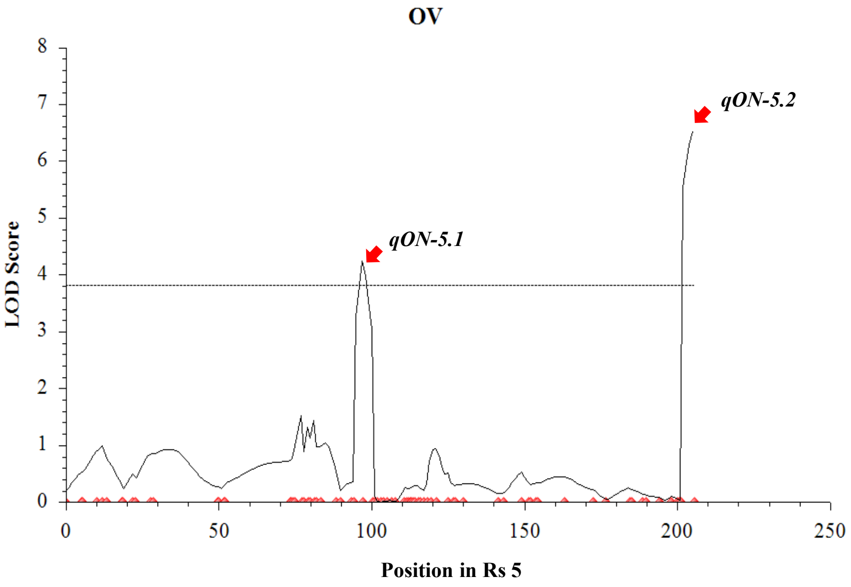

Supplemental Fig. 5 QTL mapping results on Rs 5 in 2022. Red dots on the x-axis represent the distribution of markers, red arrows indicate the signals of *qON-5.1* and the *qON-5.2* with undefined QTL region.

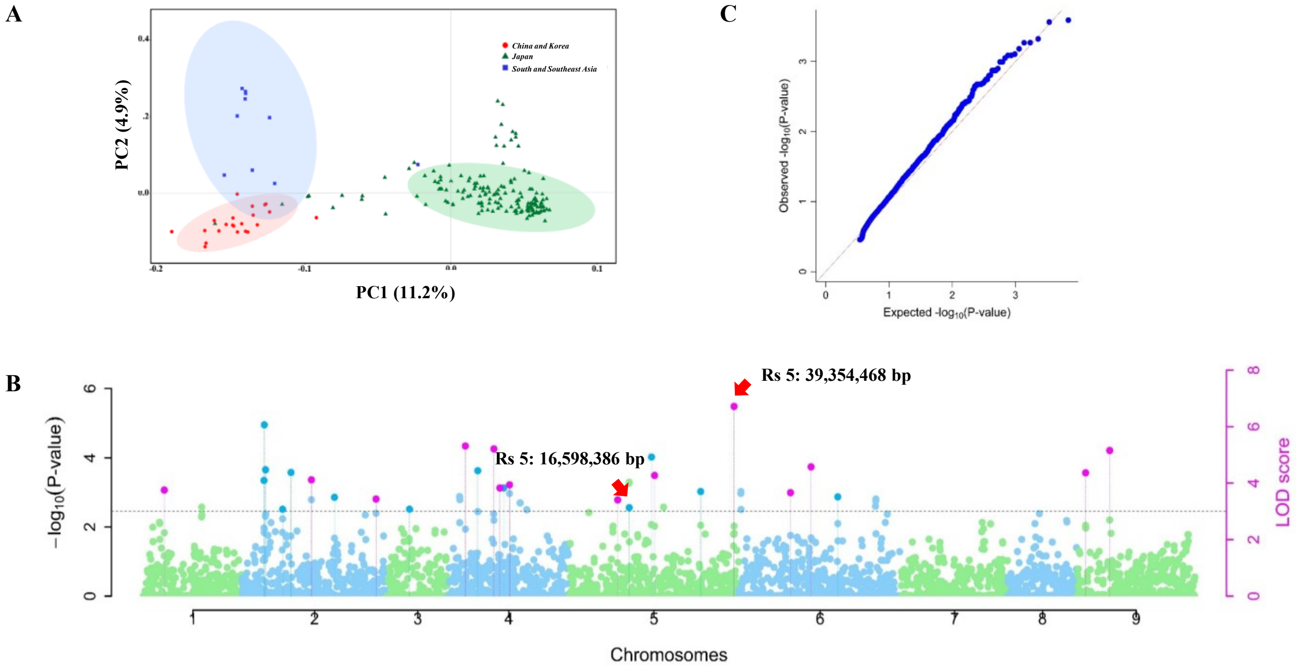

Supplemental Fig. 6 ML-GWAS results in natural population. A. Principal component analysis of natural population. B. Manhattan plot of the ML-GWAS results. The red arrow marks the detected QTNs at Rs 5: 16,598,386 bp and Rs 5: 39,354,468 bp. C. Quantile-quantile plot for ONPO-associated SNPs detected by ML-GWAS method.

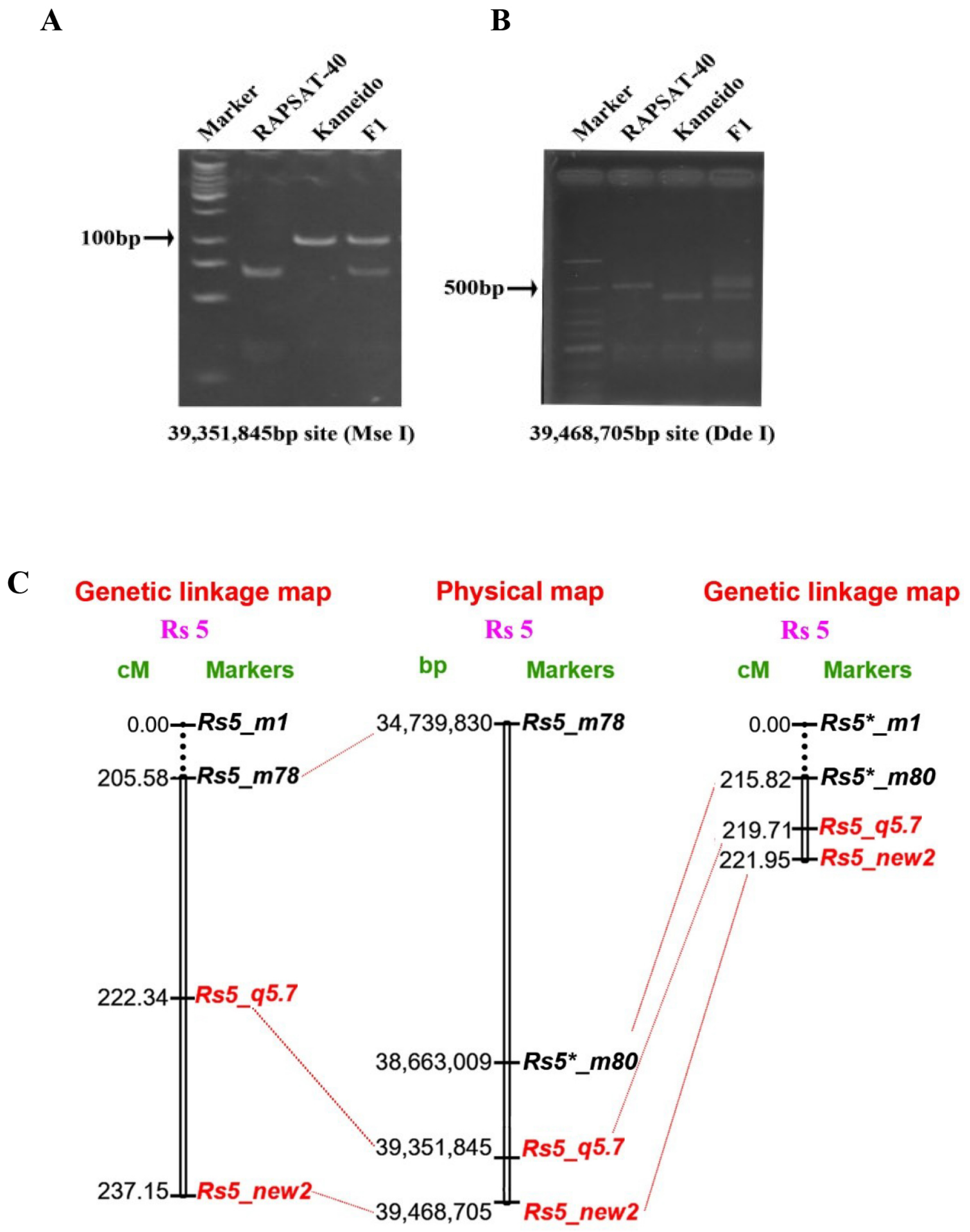

Supplemental Fig. 7 Development of PCR-RFLP markers based on GWAS results. A. Polymorphic banding patterns of PCR products digested with *Mse I* at the q5.7 site in the parental lines and their F_1 hybrid. B. Polymorphic patterns of PCR products digested with *Dde I* at Rs 5: 39,468,705 bp in the parental lines and their F_1 hybrid. C. Extended linkage group 5 after incorporating q5.7-derived marker at 39,351,845 bp and Rs5-new2 marker at 39,468,705 bp. Rs5-m1 and Rs5-m78, Rs5*-m1 and Rs5*-m80 represent the first and last markers on Rs 5 in the original genetic maps from 2022 and 2023, respectively.

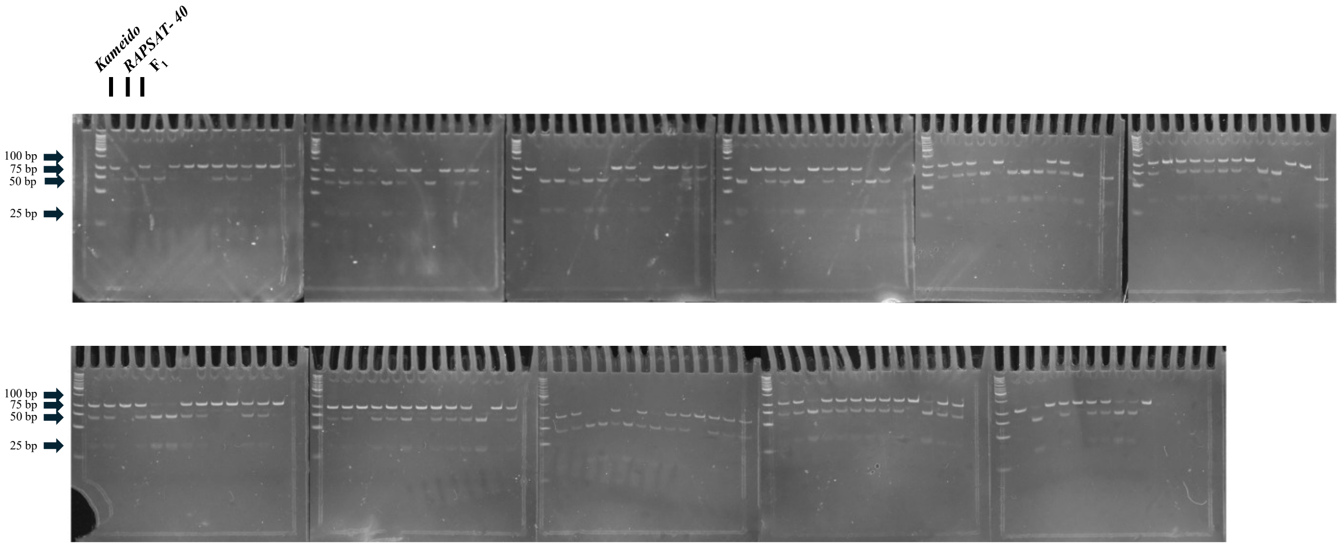

Genotype	Number
Kameido	32
Heterozygote	68
RAPSAT-40	33
Miss	1
	134

Supplemental Fig. 8 Genotyping of *q5.7* in the 2022 F_2 population. PCR products were digested with *MseI* to distinguish alleles from ‘Kameido’, ‘RAPSAT-40’, and heterozygotes. Genotyping results of 134 progenies, with genotype frequencies summarized in the accompanying table.

A

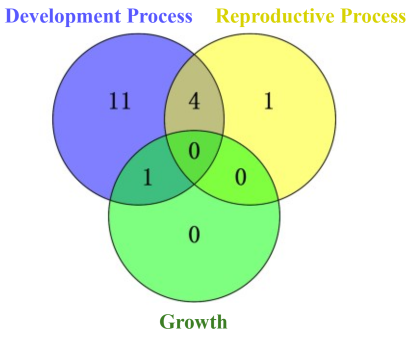

B

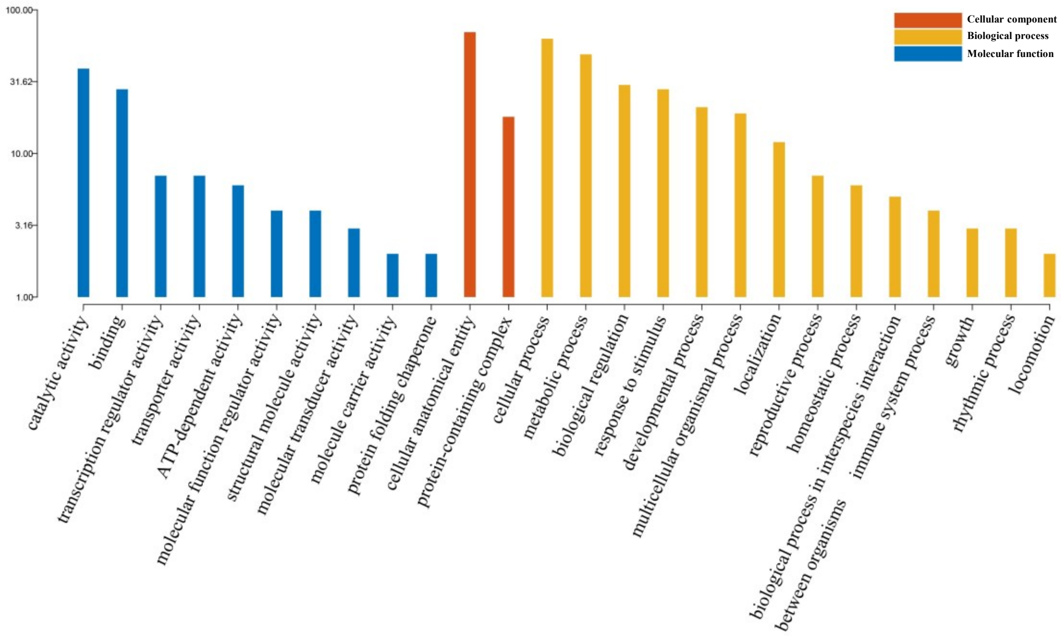

Supplemental Fig. 9 GO analysis of annotated genes within the overlapping regions on Rs 5. A. Venn diagram illustrating the number of annotated genes associated with development process, reproductive process, and growth. B. GO classification of the annotated genes at level 2.

A

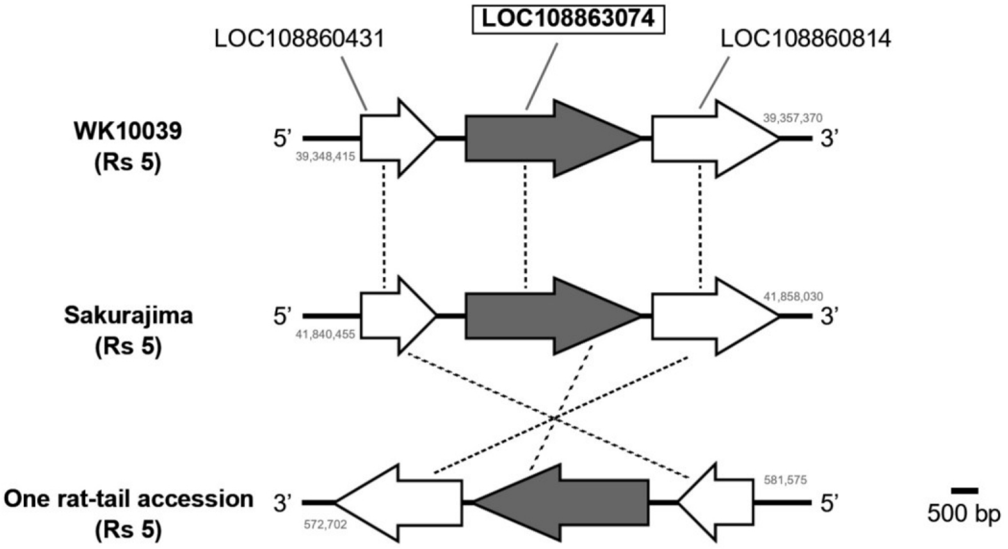

B

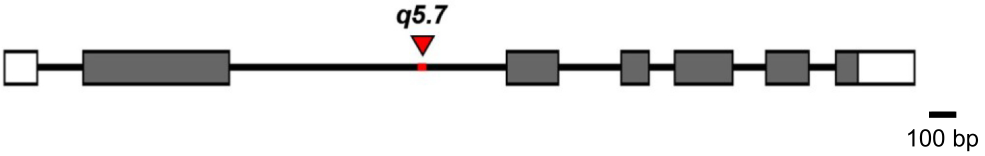

Supplemental Fig. 10 Synteny and gene structure of *RsFLK* in radish. A. Synteny of LOC108863074 (*RsFLK*) among three radish accessions. LOC108860431 and LOC108860814 represent annotated genes adjacent to *RsFLK*. B. Gene structure *of RsFLK*. The red arrow indicates the position of the *q5.7* site.

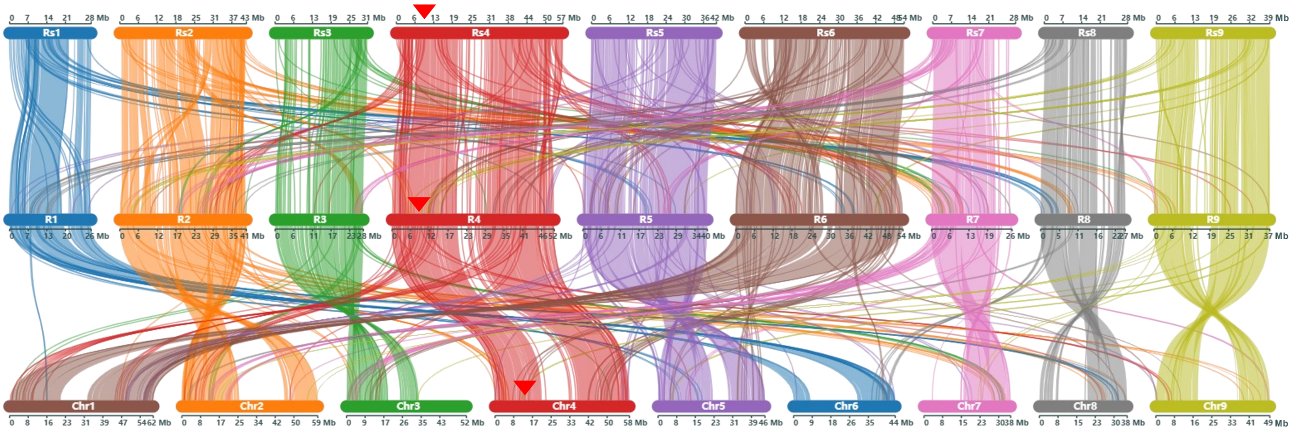

Supplemental Fig. 11 Genome synteny analysis between three represent radish genomes assembled from Japan, Korea, and China. Rs 1 to Rs 9, R 1 to R 9 and Chr 1 to Chr 9 are pseudomolecule sequences allocated as Japan, Korea, and China, respectively. The red triangles indicate the approximate positions of the orthologous gene *NERDI* in the radish genome.
